# How are treatment decisions for myocardial infarction made in the presence of advanced kidney disease? A qualitative study in the UK

**DOI:** 10.1136/bmjopen-2025-106617

**Published:** 2025-10-20

**Authors:** Jemima Scott, Lucy E Selman, Fergus J Caskey, Thomas Johnson, Yoav Ben-Shlomo, Matthew P Graham-Brown, Phillippa K Bailey

**Affiliations:** 1 Population Health Sciences, University of Bristol, Bristol, UK; 2 Richard Bright Renal Unit, North Bristol NHS Trust, Bristol, UK; 3 University of Bristol School of Social and Community Medicine, Bristol, UK; 4 Bristol Heart Institute, University Hospitals Bristol NHS Foundation Trust, Bristol, UK; 5 Department of Renal Medicine, University Hospitals Leicester, Leicester, UK; 6 Department of Cardiovascular Sciences, University of Leicester, Leicester, UK

**Keywords:** Myocardial infarction, Chronic renal failure, Clinical Decision-Making, Qualitative research, Patient-Centered Care

## Abstract

**Abstract:**

**Objectives:**

To understand why patients with chronic kidney disease (CKD) may not be treated according to international guidelines for myocardial infarction (MI).

**Design:**

Multicentre qualitative interview study. Interviews were analysed using reflexive thematic analysis approach as outlined by Braun and Clarke to generate themes associated with MI treatment decision-making for, and by, patients with CKD.

**Setting:**

Four National Health Service hospital centres in the UK (February 2022 to July 2024).

**Participants:**

A purposive sample of 46 participants (patients and clinicians). Clinicians (n=32) were senior doctors-in-training or consultants in cardiology, nephrology, acute or emergency care or cardiac surgery. Patient participants (n=14) had CKD, defined as an estimated glomerular filtration rate <60 mL/min/1.73 m^2^, or receipt of kidney replacement therapy (KRT).

**Results:**

Despite expressing strong views regarding their health priorities, patients reported minimal involvement in treatment decision-making. Decision-making by clinicians was driven by the desire to avoid causing harm to patients by ‘active’ treatment initiation. In general, despite the concept of evidence-based medicine being widely accepted, there remained scepticism of guidelines or epidemiological data, especially in the light of personal adverse experiences or anecdotes. Clinicians described how, in the absence of collaborative decision-making and a clinical safety-net for managing treatment complications, they tended to make conservative treatment decisions for patients with CKD.

**Conclusion:**

Interventions to foster teamworking between specialists and ensure adequately resourced specialist clinical service safety-nets may improve access to treatments for MI for people with CKD. Intervention development and evaluation should follow to determine if outcomes for people with CKD and MI can be improved.

**STRENGTHS AND LIMITATIONS OF THIS STUDY**

- Multi-centre design and successful recruitment of diverse participants, resulted in a rich, novel dataset, investigating myocardial infarction treatment decision-making in chronic kidney disease from multiple perspectives.
- Findings may not be transferrable to health systems beyond the UK National Health Service.
- Clinicians reflected on previous and hypothetical treatment decisions, which may not accurately reflect real-time decision-making.
- Other groups of clinicians contribute to decision-making who were not included in our study, such as general practitioners.

## Introduction

 Chronic kidney disease (CKD) is common among people experiencing myocardial infarction (MI; a ‘heart attack’) and is associated with increased mortality, morbidity and healthcare costs.^1^,^3^ However, international observational studies have shown that people with CKD are less likely to receive cardioprotective medications or invasive revascularisation procedures, post MI, than those without kidney disease.^2 4^ Such variation in care impacts not only individuals experiencing non-ST elevation MI (non-STEMI) but also those with STEMI for whom highly protocolised emergency intervention is advocated within international guidelines.^4^,^7^

People with CKD have been excluded from most large-scale randomised controlled trials that have advanced MI care over the past 50 years.^8^ Our knowledge of optimal care for this population is therefore limited. Where guidance does exist, observational research suggests it is not being followed.^4 9^ We do not know how treatment decisions are made in the absence of high-quality evidence. It is unclear what factors influence decision-making, to what extent decision-making is shared, whether decisions are being driven by patient wishes, and what factors explain the reduced access to cardiac intervention for people with CKD.

This qualitative study was designed to understand how MI treatment decisions are made by people with CKD and the clinicians who care for them. It constitutes the first step towards improving treatment decision-making in this population and may contribute to the development of interventions to reduce mortality and morbidity following MI for the high-risk CKD population.

## Methods

Ethical approval for this study was granted by the National Health Service (NHS) Research Ethics Committee Southwest (Reference 21/SW/0162). The study protocol has been published.^10^

### Study design and participants

One-off, in-depth, semi-structured interviews were undertaken with:

- people with CKD who had experienced an MI, and
- clinicians with self-reported experience of managing MI in the presence of kidney disease.

### Participant recruitment

Participants were recruited from four UK NHS hospital trusts (centres), selected to represent variation in patient demographics and clinical expertise. All hospitals had inpatient cardiology services and offered percutaneous coronary intervention services at least within working hours. Three hospitals also hosted nephrology departments and two offered cardiac surgery.

Full participant inclusion and exclusion criteria are listed in online supplemental e. table 1 and summarised here.

Patient inclusion criteria: patients were required to have CKD (defined as estimated glomerular filtration rate (eGFR) <60 mL/min/1.73 m^2^ or receiving KRT) and to have been hospitalised for an MI in the preceding 2 years. Patients were purposively selected, aiming for diversity regarding ethnicity, gender, socioeconomic status, age and receipt of KRT.

Clinician inclusion criteria: clinician participants were senior doctors-in-training (‘registrars’) or consultants in cardiology, nephrology, acute or emergency care or cardiac surgery. Clinicians were purposively selected, aiming for variation in clinical specialty, age, gender and ethnicity.

Participants were given written information regarding the study at invitation and provided written consent to participate. Further information regarding recruitment is presented in online supplemental e. table 2.

### Data collection

Semi-structured interviews were conducted by JS.^11^ Interviews were undertaken in person or by video conference or telephone according to participant preference, and audio-recorded.^12^ Flexible topic guides were developed for patient and clinician interviews (online supplemental e. table 3 and 4)**,** based on study aims, literature review and input from a patient advisory group. Demographic data were collected at the time of the interview. Recruitment ceased when the researchers (JS/LES/PKB) deemed that sufficient information power had been attained.^13^

### Data analysis

Interviews were transcribed verbatim prior to analysis according to the six steps of reflexive thematic analysis as outlined by Braun and Clarke, taking a social constructionist position.^14^ Inductive coding was undertaken by assigning descriptive labels to sections of text to highlight semantic or latent meaningfulness. Codes were collated and organised into clusters with shared meaning relating to a central concept (‘themes’).^15^ Data from patient and clinician participants were analysed separately, and the findings combined by identification of overarching themes which captured both coding frameworks. JS undertook coding, using NVivo V.14 for data management. LES, PB and patient advisors participated in refinement and sense-checking of themes. Researcher reflexivity is described in online supplemental e. table 5.

The study is reported according to the Consolidated Criteria for Reporting Qualitative Research framework (online supplemental e. table 6).^16^

### Patient and public Involvement

A mixed-methods programme of work relating to equity of access to care for MI was designed following consultation with the UK Renal Registry Patient Advisory Group; cardiovascular care was identified as a priority area. This manuscript reports the qualitative study component. Following receipt of funding, a six-member patient group was developed comprising individuals with experience of both kidney and cardiovascular disease. Patient members contributed to the wording of all patient-facing documents, the contents of the patient interview topic guide, the patient recruitment strategy and sense-checking of themes. They will contribute to the dissemination of results to the wider patient community.

## Results

### Participants’ characteristics

Of 110 individuals invited to take part in the study (37 patients and 73 clinicians), 47 agreed to take part. One patient withdrew from the study following the interview. Participants therefore included 32 clinicians (12 cardiologists, 9 nephrologists, 8 acute and/or emergency clinicians and 3 cardiac surgeons) and 14 patients (3 with CKD not receiving KRT, 2 kidney transplant recipients, 3 peritoneal dialysis patients and 6 haemodialysis patients; tables1  2). Participant numbers by site are presented in online supplemental e. table 7. Interviews lasted approximately 1 hour (36–84 min).

**Table 1:** Characteristics of clinician participants

Characteristics	Category	N (%)
Gender	Female	11 (34)
	Male	21 (66)
Age range (years) clinicians	20–39	14 (44)
	40–49	11 (34)
	≥50	7 (22)
Ethnicity	White	20 (63)
	Black, Asian and other	12 (38)
Clinical specialty	Cardiology	12 (38)
	Nephrology	9 (28)
	Acute and emergency medicine	8 (25)
	Cardiac surgery	3 (9)
Grade	Consultant	22 (69)
	Registrar	10 (31)
Location of undergraduate medical training	UK	27 (84)
	Outside UK	5 (16)
Years working in current role	0–5	16 (50)
	5–10	8 (25)
	≥10	8 (25)
Years working in current centre	0–5	19 (59)
	5–10	6 (19)
	≥10–15	7 (22)

**Table 2:** Characteristics of patient participants

Characteristics	Category	N (%)
Gender	Female	5 (36)
	Male	9 (64)
Age range (years) patients	50–59	1 (7)
	60–69	3 (23)
	70–79	4 (31)
	≥80	6 (46)
Ethnicity	White	12 (86)
	Black, Asian and other	2 (14)
Marital status	Married or living as married	11 (79)
	Widowed partner	3 (21)
Use of kidney replacement therapy	None	3 (21)
	Kidney transplant	2 (14)
	Haemodialysis	6 (43)
	Peritoneal dialysis	3 (21)
Highest level of education	Secondary school or vocational/technical	8 (57)
	University undergraduate degree	6 (43)
Religion	Religious	5 (36)
	No practising religion	9 (64)
Employment status	Part-time employment	1 (7)
	Full-time employment	2 (14)
	Retired	11 (79)
MI therapy received	Medical therapy only	5 (36)
	Stent	7 (50)
	Bypass operation	2 (14)

MI, myocardial infarction.

### Themes and subthemes

Seven main themes were identified: (1) limited patient involvement in treatment decisions; (2) inter-clinician communication supports high-risk decision-making; (3) variation in use of written guides to decision-making; (4) the safety net of associated health services support intervention; (5) the value assigned to experience over evidence; (6) individual perception of risk and benefit; and (7) harm from action perceived as worse than inaction. Illustrative quotes are presented in online supplemental e. table 8.

#### Limited patient involvement in treatment decisions

Patients and clinicians reported that shared MI treatment decision-making was rare. Cardiologists felt this was because cardiology was historically a ‘type A (hierarchical/autocratic) specialty’ (consultant cardiologist, Trust 3) and believed their patients preferred them to make decisions. Some described involving patients more often when they were themselves in doubt about the optimal treatment (Q1). Several clinicians said spending time getting to know patients was critical for both involving them in treatment decisions and making decisions on their behalf (Q2). Most felt shared decision-making (SDM) was limited by the lack of evidence-based estimations of risk (Q3), although several described how such figures could be framed differently to influence the opinion of a patient (Q4).

Only one patient reported involvement in treatment decision-making (Q5). This individual was female and used both NHS and private medical services. Most patients described being presented with a single ‘optimal’ treatment (Q6). Patients perceived the complexity of information relating to MI management to be a barrier to their involvement in decisions (Q7). While some clinicians suggested patients were too unwell to engage in SDM, no patients reported this. However, several described how being an inpatient put them in a compromised and inferior position (Q8), and that the consultant ward round did not invite expressing opinions (Q9). Most patients denied wanting to be involved in decision-making, but reported they did want to be informed about what treatments would involve, and related risks (Q10). Simple communication strategies such as maintaining confidentiality were perceived to have been undertaken poorly, resulting in significant distress (Q11).

#### Inter-clinician communication supports high-risk decision-making

##### Collaborative nature of decisions

**Figure 1:**
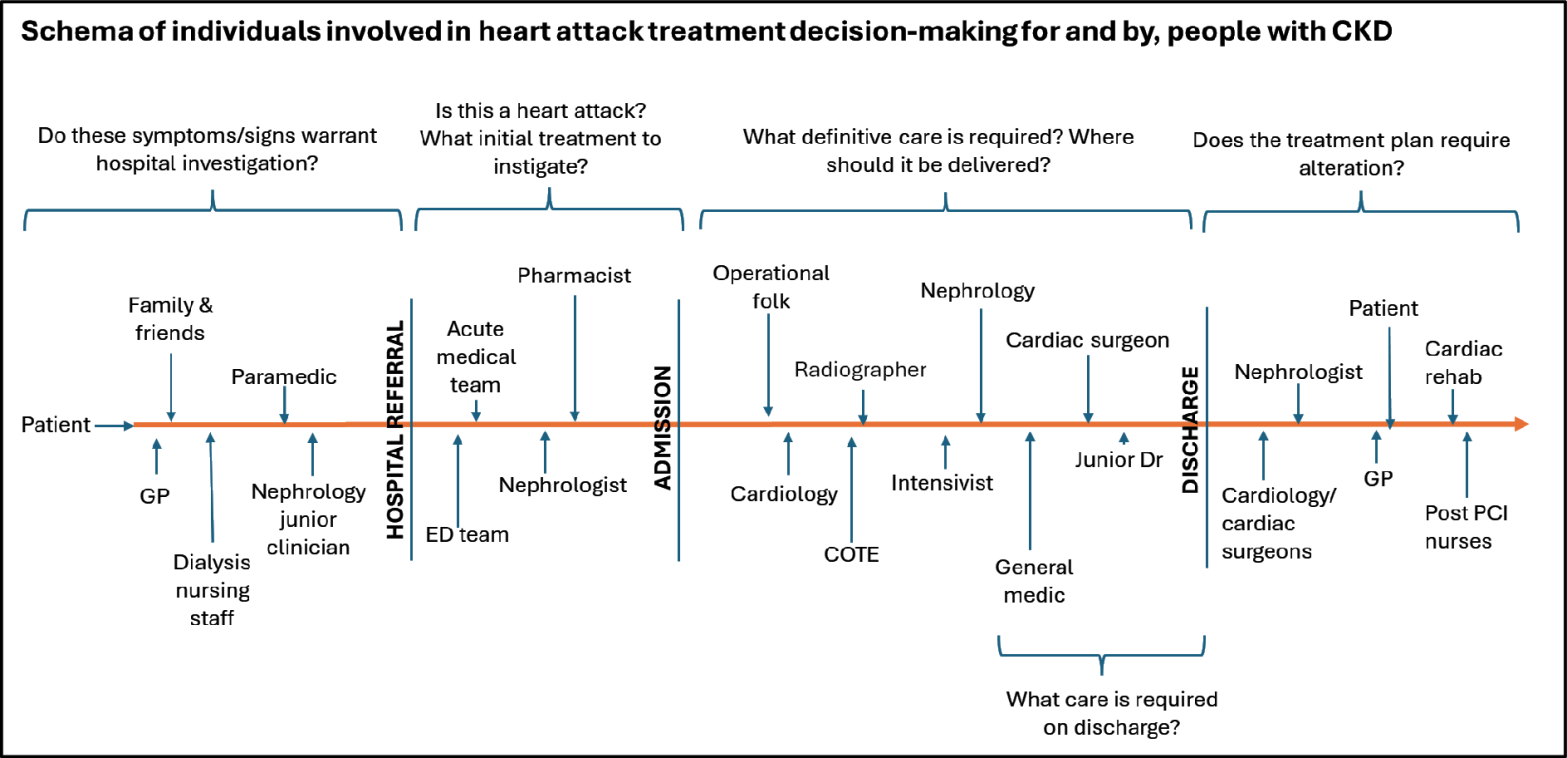
Treatment decisions and decision-makers in the myocardial infarction pathway for people with CKD.

CKD, chronic kidney disease; COTE, care of the elderly; Dr, doctor; ED, emergency department; GP, general practitioner; PCI, percutaneous coronary intervention.

Many individuals were reported to be involved in decision-making (figure 1). These extended beyond those included in this study, to the family members of patients, paramedics, nursing staff, pharmacists, radiographers, cardiac intensivists and general practitioners (GPs). Multiple decision points were reported along the treatment pathway, with several individuals involved at each.

Collaborative working between clinicians was widely believed to facilitate timely and ‘appropriate’ treatment decisions. Decision-making support from seniors and clinicians in other specialties was noted to be particularly important for high-risk decisions. Collaborative working allayed fears relating to patient harm (Q13), increased confidence and spread the psychological burden of potential adverse outcomes (Q14) and litigation. Patients described increased trust in decisions discussed with their nephrologists; some described asking for this communication to take place (Q15). One senior consultant noted, however, that multidisciplinary team meetings (MDTs) “are only as good as the people within them” (consultant cardiologist, Trust 2 (Q16)). Collaborative decision-making was described to be more common where specialists worked together within one hospital (Q17).

##### Effective relationships are dependent on trust

Trust was described as key to successful collaborative decision-making. Clinicians reported greater confidence in advice from other specialists if they were known to them, conducted in-person assessments, communicated face-to-face (Q18) and explained the reasoning underlying their treatment recommendations (Q19). One cardiac surgical registrar elaborated “[they have given] slightly more useful advice since they've been physically seeing the patients” (Trust 3 (Q20)). Consultants also highlighted the importance of trust between specialties, suggesting this was promoted by sharing a hospital site, MDT and university department (Q21). Patients expressed greater trust in senior clinicians, those who took care in discussing treatment options, or were known to them (Q22 and 23). Trust in GPs was lower than hospital specialists; they were perceived to have less in-depth knowledge (Q24).

Lack of trust between clinicians appeared to result in inappropriate and inefficient treatment decision-making (Q25). Clinicians who described poor relationships with other specialties mentioned selectively presenting patient information to influence decision-making (Q26), or repeating assessments they perceived to have been performed poorly by others. Clinicians typically described mistrust on a departmental, rather than an individual level (Q27).

### Variation in use of written guides to decision-making

Clinicians agreed that treatment guidelines influenced decision-making by junior doctors more than consultants (Q28). Regarding the interpretation of guidelines, one acute medical consultant (Trust 1) noted, “you’re much more likely not to do something if it says consider to do it, and you don’t know why you’d be making the decision to do it or not do it” (Q29). Variable trust in guidelines was also reported (Q30). Clinicians seemed to have more confidence in the verbal opinions of seniors or those working in other specialties than written documents (Q31). This was most obvious when clinicians were looking for reassurance that they would not cause harm by treatment initiation (Q32).

### The safety net of associated health services support intervention

Clinicians reported making ‘higher risk’ treatment decisions when they believed support was available for potential complications. For example, cardiac surgeons described undertaking higher-risk surgery in units with respected renal and intensive care departments (Q33). Cardiologists perceived support as the presence of a second cardiologist in theatre and an on-site cardiothoracics department (Q34). Cardiologists in Trusts 2 and 3 reported being reluctant to prescribe cardioprotective but potentially ‘nephrotoxic’ medications for CKD patients due to a lack of GP appointments at which kidney function could be monitored (Q35). In contrast, one consultant cardiologist working in Trust 1 believed follow-up by nephrologists was sufficient (Q36), and patients locally tended to agree (Q37). A consultant nephrologist in the same Trust, however, felt this was “not in [his] job role” (Q38).

### The value assigned to experience over evidence

Most clinicians described the lack of evidence regarding optimal MI management in patients with CKD as a barrier to decision-making (Q39). Despite wishing for more evidence, several clinicians appeared sceptical of the applicability of research to individual patients (Q40). Furthermore, there was variation in the interpretations of specialists of existing data, associated with differences in clinical experience. For example, while nephrologists denied seeing cases of significant kidney impairment resulting from angiographic contrast and believed the data did not support significant nephrotoxicity (Q41), surgeons described having seen such cases and reported “there are trials showing that [contrast dye is very nearly toxic]” (consultant cardiac surgeon, Trust 2 (Q42)).

Overall, clinicians appeared to value knowledge gained from clinical experience above published evidence (Q43). One cardiologist elaborated, “whatever these other papers have said… the mortality is significant” (consultant cardiologist, Trust 1 (Q44)). Knowledge derived from experience was described as established over years of practice and circulated between clinicians within, but not outside, departments (Q45).

### Individual perception of risk and benefit

#### Differential risk to organs facilitates decision-making

Clinicians perceived treatment decision-making to be easier when the risk to one organ (kidney or heart) was life-threatening (Q46 and 47). For example, few could imagine a case where a patient with a life-threatening MI would not receive invasive management, regardless of their kidney function (Q48). Similarly, clinicians agreed that invasive management was inappropriate for patients with advanced CKD receiving conservative kidney care, due to the risk to residual kidney function (and thus life; Q49). Interestingly, few patients spoke about prioritising one organ over another, even when directly asked; their focus was overwhelmingly on maintaining their independence (Q50).

#### Influence of kidney replacement therapies on perceived risks

The (planned) receipt of KRTs influenced decision-making by all participants. For many clinicians, the availability of KRTs encouraged them to prioritise the heart above the kidney (Q51). Individuals with or awaiting a kidney transplant were thought appropriate for invasive management as they had been deemed fit for surgery and required optimal cardiac function to survive this (Q52). Patients suspended from the transplant list indicated they would undergo any intervention to be relisted (Q53). In contrast, perceptions of the ‘meaning’ of dialysis differed. Some believed dialysis represented ‘active’ therapy, suggesting these patients should receive invasive MI management (Q54). Conversely, a few non-nephrologists equated receipt of dialysis with significant frailty and thus suitability for medical MI management only (Q55). One patient believed clinicians considered all CKD patients “on [their] way out” (Q56).

### Harm from action perceived as worse than inaction

Most clinicians spoke of concerns regarding patient harm. When clinicians spoke of their own decision-making, they tended to focus on the risks associated with treatment (Q57). In contrast, when discussing decisions made by others, the focus was more often on harm from treatment delays or omissions (“it’s often a delay and a reluctance to act” (nephrology registrar, Trust 1 (Q58))).

Several clinicians reported feeling more concerned about causing harm via treatment than that arising through inaction (Q59). Many described harms relating to treatment as their *fault* (“every percent off that eGFR we’re going to be killing them” (consultant cardiologist, Trust 2 (Q60))). Memorable cases with negative outcomes were reported to have substantial impact on the psychological health of clinicians (Q61). The perceived potential to *cause* a person to require dialysis was described as a particular emotional burden by non-nephrologists (Q62).

Clinicians attributed blame for treatment delays and omissions mostly to individuals outside their own specialty. For example, acute and emergency physicians perceived lack of direct communication between medical specialists to be the primary cause of treatment delay (Q63). Nephrologists from Trust 1 believed excessive use of non-invasive investigations by cardiologists was to blame (Q64), and spoke of decision-making by cardiologists and surgeons for patients with CKD as overly conservative. Cardiologists and cardiac surgeons were described as attempting to persuade one another to accept higher-risk patients (Q65).

**Figure 2:**
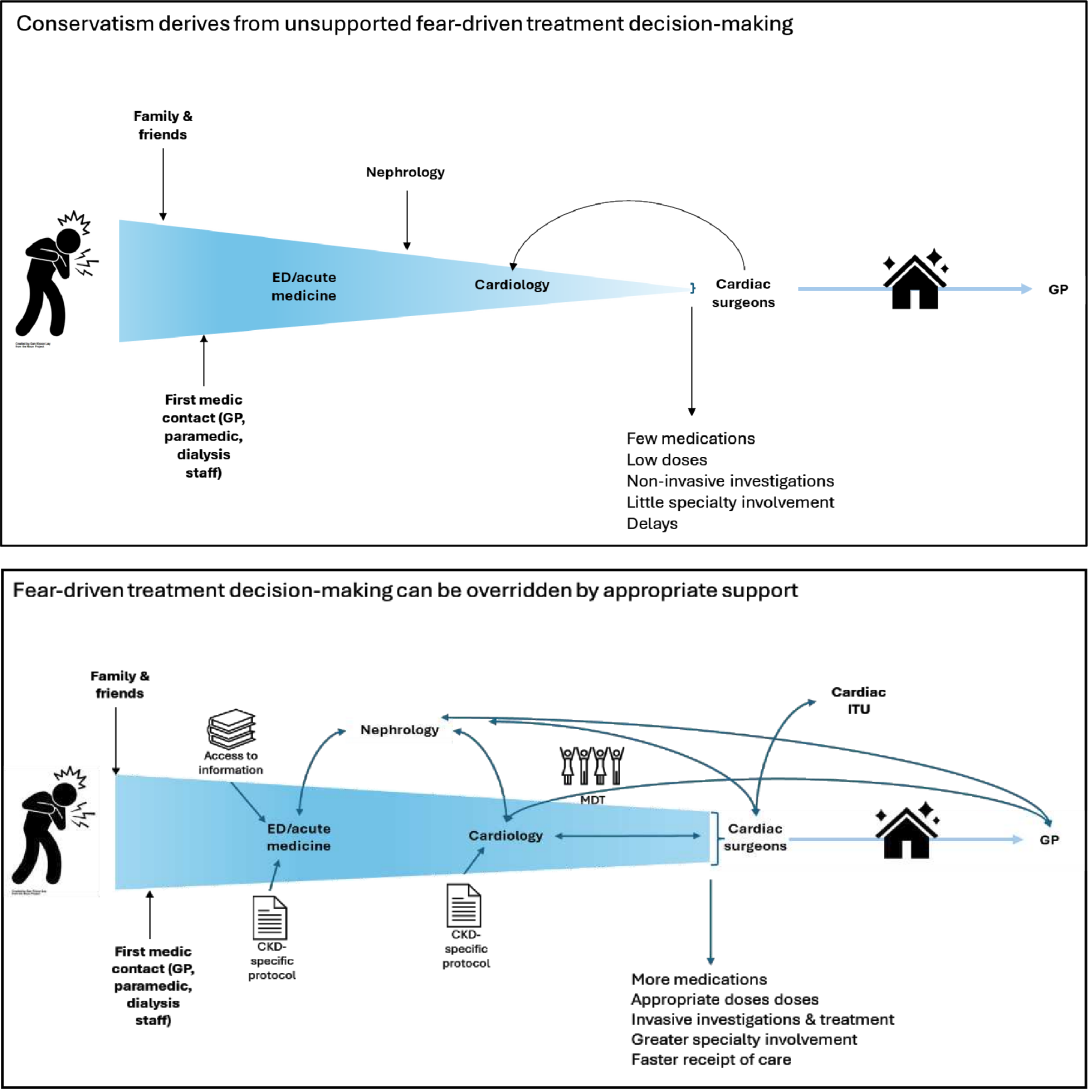
Fear of patient harm from active treatment drives conservatism in treatment decision-making.

Thematic schema of the described themes is shown in figures2  3.

Pathway by which fear of patient harm arising through active treatment results in a bias towards conservative myocardial infarction treatment decisions (panel 1), which could be overcome by support for treatment decision-making and safety-netting of potential complications (panel 2).

CKD, chronic kidney disease; ED; emergency department; GP, general practitioner; ITU, intensive therapy unit; MDT, multidisciplinary team.

**Figure 3:**
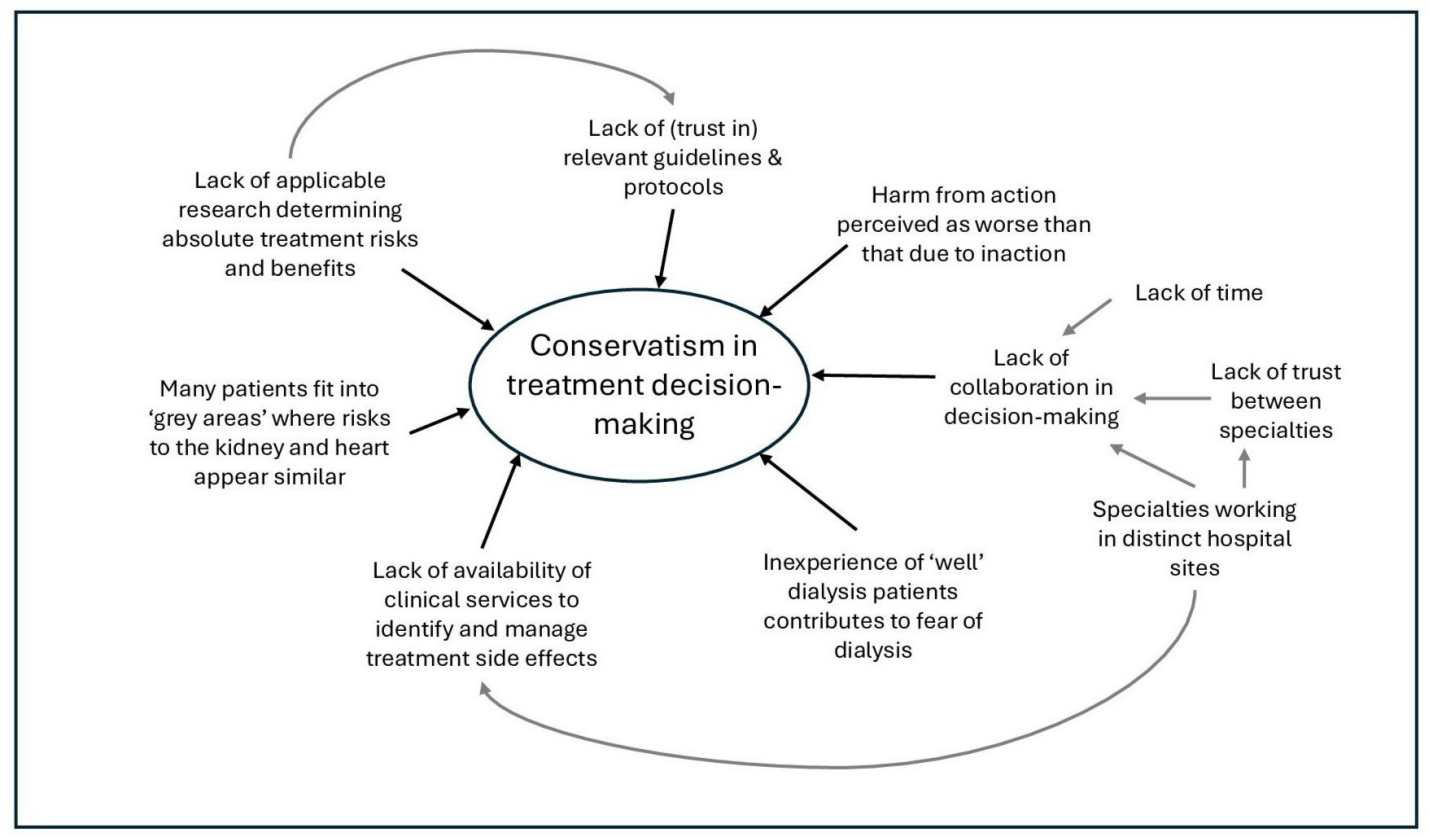
Factors contributing towards conservatism in heart attack treatment decision-making for people with chronic kidney disease.

## Discussion

To our knowledge, this is the first qualitative study to explore both the factors influencing MI treatment decisions in CKD, and the process of decision-making. Patients held strong treatment preferences, and yet their involvement in treatment decisions was minimal. Decisions of clinicians were driven by the desire to avoid patient harm from MI therapies. The potential to ‘cause’ kidney failure was the most feared outcome among non-nephrologists; however, in general, differences in treatment decision-making appeared more marked between individual clinicians than between specialties. Effective collaborative working and the availability of clinical services to treat potential complications were perceived to enable clinicians to overcome a bias towards conservative treatment decisions for the CKD population.

Minimal patient involvement in treatment decision-making was observed in this study. This is comparable to the findings of qualitative research in Canada regarding acute cardiac care in CKD and non-CKD populations.^17 18^ Similar barriers to SDM were identified in both studies: the urgency of decisions, complexity of information and how unwell patients felt.^17 18^ In addition, patients in this study highlighted that the clinician’s manner and the process of the hospital ward round inhibited effective communication between them and their clinical team. Only one individual (female, receiving private medical care) described active involvement in treatment decision-making. Despite this, all patients held strong views about their health goals.

Clinicians reported involving patients most often in decisions where they felt there to be clinical equipoise. Many perceived these to be the most challenging discussions, however, due to lack of evidence on treatment risks and benefits. In general, despite the concept of evidence-based medicine being widely accepted, there remained scepticism of guidelines or epidemiological data, especially in the light of personal adverse experiences or anecdotes. This highlights the challenges in translating evidence into real world clinical management. It remains to be seen, therefore, whether the updated recommendation in the 2023 European Society of Cardiology guidelines to ‘apply the same diagnostic and therapeutic strategies (for MI) in patients with CKD as in patients without kidney disease’ will significantly alter clinical practice.^19^

Clinicians in this study identified multiple factors that steered them towards conservative MI treatment recommendations for patients with CKD. The most significant was the drive to avoid patient harm. Clinicians described high levels of personal responsibility and self-blame for negative treatment outcomes, even where these were well-recognised treatment side effects. Feelings of guilt and shame have been described in previous qualitative work on physicians and their experiences of medical errors^20^ and found to contribute to depression and burnout.^21 22^ To our knowledge, the psychological burden of treatment side effects has not been investigated.

Clinicians described perceiving harm from active MI treatment to be more blameworthy than harm secondary to undertreatment. Such ‘omission’ bias was first described with reference to vaccination uptake, whereby parents would decline vaccinating their children to avoid a small risk of complications, despite a much greater risk of harm if unvaccinated.^23^ It has since been described in numerous other situations.^24^,^26^ In this study, some clinicians were aware of their own bias towards omissions. It is unclear whether such knowledge enabled them to overcome this when making treatment recommendations.

Given the high perceived responsibility for treatment outcomes, it is unsurprising that clinicians described making higher-risk decisions when they felt supported in decision-making. The most highly regarded support was collaborative working between clinicians. The efficacy of this, however, depended on trust. While clinicians appeared to have inherent trust for those within their own specialty, trust for those from other specialties needed to be earned. This concept of ‘us versus them’ with regards to interspecialty boundaries was described in a qualitative study of interphysician communication in the USA.^27^ Boundaries between emergency and internal medicine physicians were perceived to prime these specialties for conflict.^28^ Clinicians in both studies described how face-to-face working could, however, overcome these barriers and increase mutual trust.^27^

Variation in treatment decision-making appeared greater between individual clinicians than between specialties. Specialty did appear, however, to influence decisions regarding: (i) estimation of prognosis of individuals with CKD (non-nephrologists appeared more pessimistic); and (ii) interpretation of the evidence base. Despite access to the same body of published research, the views of clinicians on clinical concerns such as the risk and impact of contrast-associated nephropathy varied widely between specialty. As suggested previously, it is possible that clinical experiences (personal and those of trusted colleagues) influence clinicians in their choice and interpretation of research papers.

Study strengths are the multicentre design and successful recruitment of diverse participants, producing a rich, novel dataset, investigating MI treatment decision-making in CKD from multiple perspectives. However, findings may not be transferrable to health systems beyond the UK NHS. Clinicians reflected on previous and hypothetical treatment decisions, which may not accurately reflect real-time decision-making. Patient participants were predominantly those receiving KRTs. Some of the themes, such as ‘Effective relationships are dependent on trust’ may be less relevant to time-critical care such as that for STEMI. Other groups of clinicians who contribute to MI decision-making were not included in our study, such as GPs. Future research should focus on the barriers and facilitators to MI decision-making in CKD experienced by these clinicians.

This multicentre qualitative study identified a divorce between the strength of healthcare preferences held by CKD patients and their involvement in treatment decision-making. Unaware of these preferences, clinicians were inclined to make conservative treatment decisions on behalf of those with CKD. Adequate inter-clinician support for decision-making and the existence of a safety net for potential treatment complications were reported to counter this bias. These findings help to explain why observational studies find MI care for those with CKD to be more conservative than treatment guidelines recommend and highlight the need to amplify the patient voice in complex healthcare decision-making.

## Supplementary material

10.1136/bmjopen-2025-106617 online supplemental file 1

## Data Availability

Data are available upon reasonable request.
