# Supplementary material for: How are treatment decisions for myocardial infarction made in the presence of advanced kidney disease? A qualitative study in the UK
Source: BMJ Open. 2025 Oct 20;15(10):e106617. doi: 10.1136/bmjopen-2025-106617 (PMC12542582; doi:10.1136/bmjopen-2025-106617)
Supplement: online supplemental file 1 [file bmjopen-15-10-s001.docx]

**Electronic file**

Contents

[**e.Table 1. Participant inclusion and exclusion criteria and method of recruitment** 2](#_Toc205801674)

[**e.Table 2. Participant recruitment and interview details** 3](#_Toc205801675)

[**e. Table 3. Example flexible topic guide - clinical staff topic guide V2.0** 4](#_Toc205801676)

[**e. Table 4. Example flexible topic guide - patient topic guide V2.0 23.11.2021** 6](#_Toc205801677)

[**e.Table 5. Reflexivity – details of the Chief Investigator** 8](#_Toc205801678)

[**e.Table 6. COREQ (COnsolidated criteria for REporting Qualitative research) checklist** 9](#_Toc205801679)

[**e.Table 7. Number (%) of participants recruited from each Hospital Trust** 11](#_Toc205801680)

[**e. Table 8. Illustrative Quotations** 12](#_Toc205801681)

## **e.Table 1. Participant inclusion and exclusion criteria and method of recruitment**

| **Eligibility criteria** | **Participants** | |
| --- | --- | --- |
|  | **Patients** | **Clinical staff** |
| **Inclusion** | UK-resident adults aged ≥18 years with moderate to severe kidney disease (CKD stages 3-5, +/- kidney replacement therapy) who have received treatment for a heart attack in a UK hospital within the past 24 months | Doctors and nurses currently practising medicine in the UK, who have self-reported experience of managing heart attacks in people with kidney disease (CKD stage 3-5, on kidney replacement therapy). |
| **Exclusion** | Patients with kidney disease who:   - Were deemed by their clinical team to be lacking the mental capacity to consent to involvement in the study. - Underwent coronary valve or aortic root repair or replacement during the MI hospitalisation. | N/a |
| **Recruitment method** | - Study information was mailed to potential participants | - Study information was emailed to potential participants |

CKD; chronic kidney disease, MI; myocardial infarction, UK; United Kingdom

## **e.Table 2. Participant recruitment and interview details**

|  | **Participants** | |
| --- | --- | --- |
|  | **Patients** | **Clinical staff** |
| **Recruitment method** | - Study information was mailed to potential participants | - Study information was emailed to potential participants |
| **Number of drop-outs** | - 1 participant withdrew from the study due to lack of consistency between verbal and written consent regarding reading of the study information | - No participants withdrew |
| **Presence of individuals other than interviewer and participant** | - Family members were allowed to be present | - Other clinicians were allowed to be present |
| **Field notes** | - Minimal field notes were made to limit disruption to in-person interviews | - Field notes were made |
| **Transcript checking** | - Transcripts were not returned to participants for comment | - Transcripts were not returned to participants for comment |

## **e. Table 3. Example flexible topic guide - clinical staff topic guide V2.0**

| **Topic guide – clinical staff** | |
| --- | --- |
| Check consent |  |
| **Experiences of MI care for people with CKD** | Could you tell me how you have been involved in looking after people with chronic kidney disease who have had a heart attack? |
|  | Prompts:   - How often do you look after these sorts of patients? - What is your specific role? |
| **CKD pathway** | Could you describe the journey of a patient with CKD through the hospital after admission with a heart attack, and whether this differs from someone without kidney disease? |
|  | Prompts:   - Which department(s) do they typically present to? - Which team(s) look after people with CKD after a heart attack in your hospital? - Are there differences in the ward(s) they are admitted to? The specialists involved. How are they discharged? |
| **Challenges of CKD** | Is the treatment of heart attack different for people with chronic kidney disease? In what ways? |
|  | Prompts:   - Why is treatment different? - What are the most important differences? |
| **Challenges of decision-making** | What are the most important decisions that you are faced with when treating heart attacks in people with kidney disease? |
|  | Prompts:   - Are these decisions different to ones that you might make when treating someone without kidney disease? - Are they difficult to make? - [If difficult] Why? - [if difficult] Which are the hardest? - [If difficult] What worries you the most? - How long do these decisions take? |
| **Process of decision-making** | How are these decisions made? |
|  | Prompts:   - Who is involved/ who is the primary decision-maker? - Is it easy to contact/bring together people involved? - Do you ever have disagreements within the team? - Are the patient/their family involved? - What sources of information do you use? How useful are they? - Do you use guidelines? What do you think of them? - What would help to make these decisions more easily? |
| **Communicating with patients** | How much involvement do you find patients want in decision-making? |
|  | Prompts:   - What information do they want/need? - How do they get information? - [If involved in delivery of info] How do you tell them about the risks and benefits of different treatment options? - Does the patient ever disagree with the clinical team? |
| **Provision of care** | Once treatment decisions have been made, are there any problems *delivering* care to people with kidney disease? |
|  | Prompts:   - [If so] Are these specific to people with kidney disease? - Do you have the facilities and staffing that you need? - Can you think of anything that would make delivering good care to people with kidney disease easier? |

CKD; chronic kidney disease, MI; myocardial infarction

## **e. Table 4. Example flexible topic guide - patient topic guide V2.0 23.11.2021**

| **Topic guide – Patient interviews** | |
| --- | --- |
| Check consent | |
| **Kidney disease** | Could you tell me a little about your kidney disease and the treatment you receive for it? |
| **Experiences of heart disease** | How did you first become aware of your heart issues?   - [If not mentioned] Were the problems with your heart diagnosed before or after your kidney condition |
|  | [If not the same event as above] Could you describe what happened to you and how you felt when you had your heart attack?  *(Prompts: What did it feel like, how and when did you get help?)* |
|  | Do you have ongoing heart problems?   - [If yes] How do these affect you?   *(Prompt: Do you struggle physically because of your heart?)* |
| **Experiences of care** | Could you describe your care in hospital? |
|  | Do you know what type of ward you stayed on? |
|  | Do you know what type of doctors you met when in hospital? For example, heart or kidney specialists. |
|  | Do you think your kidney disease affected your care in hospital or any follow-up care you received? How? |
| **Treatment decision-making** | Were you aware of any decisions being made what treatment you should receive?  *(Prompt…. for example, choices of tablets or procedures?)* |
|  | [If YES] Who was involved in making this/these decision(s)? |
|  | [If YES] Were you involved in these decisions? In what ways? |
|  | [If YES] Were you given information to help you to make your decision?   - [If NO] Do you think it would have been helpful to have had some more information? - [If YES] Could you describe how the information was given to you, and how useful you found this? - How would you prefer to receive information about your heart, and by whom? |
|  | How did you make your decision(s)? What were the most important things for you to think about? |
|  | Can you think of anything that would have helped you when making these decisions? |
| **Understanding of heart disease** | Before you started to have problems with your heart, did you know you were at risk of heart disease?   - [If YES] How did you know this? |
|  | Was it/would it have been helpful to have known that you might develop heart problems?   - [If YES] How do you think you would like to be given this information? |
| **Health priorities** | How would you describe your daily life living with heart and kidney disease?  *(Prompt: Does this affect what you are able to do each day?)* |
|  | Do you worry about your health in the future? |
|  | Do these worries affect how you live your daily life/your enjoyment of your life? |
| **Covid** | Do you think the COVID-19 pandemic has influenced how your heart disease has been managed over the past two years? In what ways? |
|  | Do you think the COVID-19 pandemic has influenced your priorities in terms of your health? |

## **e.Table 5. Reflexivity – details of the Chief Investigator**

| Current job role | Trainee nephrologist working in a UK hospital with tertiary kidney and secondary cardiology services. This work was undertaken as part of her Doctoral Fellowship. |
| --- | --- |
| Previous relevant experience | - Prior clinical experience of making MI treatment decisions for, and with, people with and without CKD. - Broad understanding of the quantitative observational evidence base demonstrating variation in MI care between these populations. |
| Research interests | Equity of utilisation of healthcare services and understanding treatment variation. |
| Theoretical sensitivity and reflexivity during the research process | - Input from a patient advisory group - Extensive review of the relevant literature - Maintaining a research diary - Regular meetings with team members (PB and LS) for reflexive discussion |
| Participant knowledge of the interviewer | - The Chief Investigator’s background and reasons for doing the research were described to potential participants in the written participant information. |

CKD; chronic kidney disease, MI; myocardial infarction, UK; United Kingdom.

## **e.Table 6. COREQ (COnsolidated criteria for REporting Qualitative research) checklist**

| **Topic** | **Item No.** | **Guide Questions/Description** | **Reported on**  **Page No.** |
| --- | --- | --- | --- |
| **Domain 1: Research team**  **and reﬂexivity** | | | |
| *Personal characteristics* | | | |
| Interviewer/facilitator | 1 | Which author/s conducted the interview or focus group? | 6 |
| Credentials | 2 | What were the researcher’s credentials? E.g. PhD, MD | 1 |
| Occupation | 3 | What was their occupation at the time of the study? | e. Table 5 |
| Gender | 4 | Was the researcher male or female? | e. Table 5 |
| Experience and training | 5 | What experience or training did the researcher have? | e. Table 5 |
| *Relationship with*  *participants* | | | |
| Relationship established | 6 | Was a relationship established prior to study commencement? | e. Table 5 |
| Participant knowledge of  the interviewer | 7 | What did the participants know about the researcher? e.g. personal  goals, reasons for doing the research | e. Table 5  X |
| Interviewer characteristics | 8 | What characteristics were reported about the inter viewer/facilitator?  e.g. Bias, assumptions, reasons and interests in the research topic | e. Table 5 |
| **Domain 2: Study design** | | | |
| *Theoretical framework* | | | |
| Methodological orientation and Theory | 9 | What methodological orientation was stated to underpin the study? e.g. grounded theory, discourse analysis, ethnography, phenomenology,  content analysis | 7 |
| *Participant selection* | | | |
| Sampling | 10 | How were participants selected? e.g. purposive, convenience,  consecutive, snowball | 6 |
| Method of approach | 11 | How were participants approached? e.g. face-to-face, telephone, mail,  email | e. Table 2 |
| Sample size | 12 | How many participants were in the study? | 7 |
| Non-participation | 13 | How many people refused to participate or dropped out? Reasons? | e. Table 2 |
| *Setting* | | | |
| Setting of data collection | 14 | Where was the data collected? e.g. home, clinic, workplace | 5 |
| Presence of non-  participants | 15 | Was anyone else present besides the participants and researchers? | e. Table 2 |
| Description of sample | 16 | What are the important characteristics of the sample? e.g. demographic data, date | 7 |
| *Data collection* | | | |
| Interview guide | 17 | Were questions, prompts, guides provided by the authors? Was it pilot tested? | e. Tables 2-3 |
| Repeat interviews | 18 | Were repeat inter views carried out? If yes, how many? | 6 |
| Audio/visual recording | 19 | Did the research use audio or visual recording to collect the data? | 6 |
| Field notes | 20 | Were ﬁeld notes made during and/or after the interview or focus group? | e. Table 2 |
| Duration | 21 | What was the duration of the inter views or focus group? | 7 |
| Data saturation | 22 | Was data saturation discussed? | 6 |
| Transcripts returned | 23 | Were transcripts returned to participants for comment and/or | e. Table 2 |

## **e.Table 7. Number (%) of participants recruited from each Hospital Trust**

|  | **Participants**  **N (%)** |
| --- | --- |
| Trust 1 | 22 (48) |
| Trust 2 | 8 (17) |
| Trust 3 | 9 (20) |
| Trust 4 | 7 (15) |

## **e. Table 8. Illustrative Quotations**

| **Theme** | **Subtheme** | **Representative quotations** |
| --- | --- | --- |
| **Limited patient involvement in treatment decisions** |  | **Q1**. “Sometimes they say you just decide what's right for me and they want you to be quite autocratic and I find that a bit harder because normally if I'm having the conversation it's because I'm not sure and they can sway my decision.” *(Cardiology registrar, Trust 4)*  **Q2**. “I think the majority of people, unless you're making a conscious effort to a, get to know them a little bit and know what's important to them and then making a conscious effort to tell them all information you think is relevant to those specific circumstances, it's actually really hard to get informed consent because people are so ready to just agree with what you recommend.” *(Cardiac surgery registrar, Trust 3)*  **Q3**. “it's very hard for patients to fully comprehend that risk when we can't put numbers on it” *(Cardiac surgery registrar, Trust 3)*  **Q4.** “let us take the example of coronary angiography in the situation of GFR20 say, that the conversation to the patient may often you know without extremely careful explanation appear to be ‘if we do the test you’ll get kidney failure and if we don’t then we can give you tablets and that might be nearly as good’ and so there’ll be an obvious outcome to those because getting kidney failure sounds terrifying” *(Nephrology consultant, Trust 1)*  **Q5.** “but I'm quite a bossy boots anyway. [laugh] I'm not a wallflower. So I do demand things that they tell me everything and most doctors tell me everything so I'm glad.” *(Female patient, CKD, Trust 1)*  **Q6.** “I was told I’ll need a stent and I thought, ‘If that solves the problem I’m not gonna argue.’” *(Male patient, peritoneal dialysis, Trust 3).*  **Q7**. “I'm a confident individual, but I haven't got the confidence or the competence to be able to make medical decisions, even when have been given choices, I don't feel I've got the right background or the ability to make a decision. I'm very much in the experts’ hands.” *(Male patient, HD, Trust 4)*  **Q8**. “I mean, when you’re lying on your back in hospital you don’t always think of all these questions. They sort of come to you afterwards don’t they.” *(Male patient, PD, Trust 1)*  **Q9.** “It’s just his coming and going to each bed that I, I found almost like a comic farce with this person trotting along behind.” *(Male patient, CKD, Trust 1)*  **Q10**. “I’d like to sort of be included and told the pros and cons of everything” *(Female patient, HD, Trust 4)*  **Q11**. “he never introduced himself, he never gave me a name. The worst part, in a way, was he told me – because you’ve got the private rooms – with the door open, and he had a loud voice. I thought, ‘Well, anybody can hear what he’s saying.’” *(Female patient, HD, Trust 1)* |
| **Inter-clinician communication supports high-risk decision-making** | **Collaborative nature of decisions** | **Q13.** “And provide reassurance, actually, to the decision maker that this is a reasonable thing to do, and we’re experienced in managing patients with kidney failure and we can help from that side of things.” *(Renal registrar, Trust 1)*  **Q14.** “I think that’s incredibly important that one person doesn’t carry the entire responsibility for a complex decision and that that is shared that is really, really important, not just, *because* that, that sense checking is right in more acute situations for the wellbeing of a patient. Cos then, essentially, you’re getting a consensus of expertise rather than just one person’s individual decision.” *(Renal consultant, Trust 3)*  **Q15.** “will you please talk to my renal consultant?” *(Female patient, HD, Trust 1)*  **Q16.** “are only as good as the people within them” *(Cardiology consultant, Trust 2).*  **Q17.** “I feel like in the hospital where I go and consult, cardiology practice, they often don’t ask or if they do ask then the decision will already have been made really, you know, they’ll have already in their management plan balanced up that risk of kidney failure versus you know investigation or revascularisation and often have decided not to do it and sometimes I go in and try and encourage them” *(Renal consultant, Trust 1)* |
|  | **Effective relationships are dependent on trust** | **Q18.** “I find that these conversations over the phone sometimes are not as useful and what you want is to have that kind of… someone to come and see the patient and make that decision with you in person.” *(Cardiology consultant, Trust 4)*  **Q19.** “…all I’ve ever looked for when I’ve been training is someone to give me a good idea of their thought processes so I can understand what they’re thinking. Even if it’s not an evidence-based decision, I can follow their thought process so that it makes sense.” *(Acute & emergency medicine consultant, Trust 4)*  **Q20.** “[they have given] slightly more useful advice since they've been physically seeing the patients” *(Cardiac surgical registrar, Trust 3).*  **Q21.** “I think there’s, you know, usually quite a good relationship, it’s a small enough hospital that I know most of the cardiology consultants by face and by name” *(Renal consultant, Trust 4)*  **Q22. “**He warned me about everything. He was really good. I can't fault that doctor.” *(Female patient, CKD, Trust 1)*  **Q23. “**the guy [cardiologist] has been in the game yonks so he probably knows more than anybody else.” *(Male patient, HD, Trust 1)*  **Q24.** “if you’ll forgive the crude expression, the GPs are sort of the ‘Jack of all trades’ rather than specialists. That’s why I have more confidence in them for these particular issues.” *(Male patient, CKD, Trust 3).*  **Q25.** “I think there will probably be centres where things have worked well, and they’ve got good relationships so maybe it’s less delays in that centre. *(Acute & emergency medicine consultant, Trust 4)*  **Q26.** “[advanced CKD] would make me less likely to be selling intervention to them” *(Acute & emergency medicine registrar, Trust 1)*  **Q27.** “I think that we, to be explicit, have a DGH (District General Hospital) service for cardiology at best” *(Nephrology consultant, Trust 1).* |
| **Variation in use of written guides to decision-making** |  | **Q28.** “the [European Society] guidelines for cardiology are essentially the bible” *(Cardiology registrar, Trust 1).*  **Q29.** “you’re much more likely not to do something if it says consider to do it, and you don’t know why you’d be making the decision to do it or not do it” *(Acute medical consultant, Trust 1).*  **Q30.** “I remember consultants who say to me, ‘(name), guidelines are guidelines – guidelines aren't dogma.’” *(Cardiology registrar, Trust 1)*  **Q31.** “despite [there being] very clear guidelines to follow….many of them will also come to me to ask what is the best?” *(Acute medical consultant, Trust 2).*  **Q32.** “I think for the same reason that are behind these guidelines that in some way - despite … dealing with an emergency at that moment, an MI, an acute syndrome, thrombotic event - you are so scared about the risk of bleeding that they have (taught) you to be careful in the use of heparin in patients with impaired renal function and that's kind of the first thing.” *(Acute & emergency medicine consultant, Trust 2)* |
| **The safety-net of associated health services support intervention** |  | **Q33.** “when people come to work here...they see our intensive care department…they are more likely to take risks” *(Consultant cardiac surgeon, Trust 2).*  **Q34.** “our cardiology lab here without cardiothoracics onsite is not going to… it wouldn’t be sensible for them to take on high risk angiography if they dissect or something like that” *(Nephrology consultant, Trust 1)*  **Q35.** “the reality is I can never be sure that the patient will receive the, you know, the monitoring that they need basically to facilitate that in terms of blood test … So often my practice is not – you know I’ll be much more comfortable starting these medications if the EGFR is over 30.” *(Cardiology registrar, Trust 2)*  **Q36.** “I always trust the nephrology team that if there's a problem, let me know about it, rather than me trying to follow-up these patients”. *(Cardiology Consultant, Trust 1)*  **Q37.** “I just find that [GPs] will just say, “No, you need to speak to the hospital about that.” They’re definitely sort of, I wouldn't say scared, but they're very hands off.” *(Male patient, haemodialysis, Trust 4)*  **Q38.** “it’s not in my job role” *(Consultant nephrologist, Trust 1)* |
| **The value assigned to experience over evidence** |  | **Q39.** “…what we’ve not mentioned so far with me moaning about lots of heart doctors not doing the treatment properly, is that there’s no evidence is there, and so there needs to be some evidence.” *(Nephrology consultant, Trust 1)*  **Q40.** “People say you must have gold standard, double blind, randomised control studies to deliver the answer for that one patient. Wait a minute, that patient doesn't fit that typical study criteria or the study criteria was so loose that it’s irrelevant.” *(Cardiology consultant, Trust 2)*  **Q41.** “actually the risk of Contrast-induced nephropathy is vanishing rare, if not 0.” *(Nephrology consultant, Trust 3)*  **Q42.** “there are trials showing that [contrast dye is very nearly toxic]” *(Consultant cardiac surgeon, Trust 2).*  **Q43.** “So I think a lot of my knowledge is experiential” *(Acute & emergency medicine consultant, Trust 1)*  **Q44.** “whatever these other papers have said… the mortality is significant” *(Consultant cardiologist, Trust 1).*  **Q45.** “each unit is a bit of a silo where you have your own local knowledge” *(Consultant cardiac surgeon, Trust 2).* |
| **Clinicians’ assessments of the risk of MI versus its treatment** | **Differential risk to organs facilitates decision-making** | **Q46.** “… there is the priority approach. If somebody’s dying, it’s an emergency, you do your operation within a few hours, accepting all the implications of heart failures, because our intensivist can put the patient on haemofiltration and look after the kidneys.” *(Consultant cardiac surgeon, Trust 2)*  **Q47.** “it’s those ones in the middle, which is a big chunk of them, sometimes it’s clearly one way or the other. In which case, you know, it’s not so much of a conundrum, but there are lots of people in this middle ground where I think there are delays which... I can’t imagine they do the patient any good” *(Acute & emergency medicine consultant, Trust 4)*  **Q48**. “You deal with the consequences because what's going to kill them is their heart attack” *(Consultant cardiologist, Trust 2).*  **Q49.** “if it was a non-STEMI and the patient had brittle kidney function but weren't for dialysis, I think I’d probably have a conversation with the patient, but there would be a strong possibility that that patient wouldn't have. I’d choose not to do the angiogram of the patient” *(Cardiology registrar, Trust 2)*  **Q50.** “…if my heart packs up then so be it. If my kidneys pack up then so be it, but if my legs pack up that would affect me radically. I do not want to be dependent on other people” *(Male patient, CKD, Trust 3)* |
|  | **Influence of kidney replacement therapies on perceived risks** | **Q51.** “you’ll die if your heart doesn’t work, you don’t die if your kidneys don’t work” *(Consultant nephrologist, Trust 3).*  **Q52.** “I might have a lower threshold for intervening in those patients because what I don't want is their coronary disease to preclude them from having a transplant” *(Cardiology registrar, Trust 3)*  **Q53. “**I was trying to get back onto the transplant list because I got took off, the really big thing that's got me” *(Female patient, HD, Trust 4)*  **Q54.** “clearly for dialysis patients it makes no sense because there's no kidney left to worry about toxicity for pre-dialysis and for transplant patients. If the angiography is prognostically important from the heart condition, then the benefits outweigh the risks.” *(Nephrology consultant, Trust 1)*  **Q55.** “if you've got a haemodialysis patient, some of them equate that to frailty” *(Renal registrar, Trust 4).*  **Q56.** “I think they thought if you’re a renal patient, you’re on your way out” *(Female patient, HD, Trust 1).* |
| **Harm from action perceived as worse than inaction** |  | **Q57.** (“You end up doing an angio, and perforate [a] coronary [vessel], or give them a stroke” *(Cardiology registrar, Trust 1).*  **Q58.** “it’s often a delay and a reluctance to act” *(Nephrology registrar, Trust 1).*  **Q59.** “causing harm is always worse, well thought of as worse…the probabilities of you causing harm are really inflated in your head probably a lot of the time” *(Cardiology registrar, Trust 1).*  **Q60.** “every percent off that EGFR we're going to be killing them” *(Consultant cardiologist, Trust 2).*  **Q61.** “it can cause incredible stress, actually. …I think there’s mental health consequences” *(Consultant nephrologist, Trust 3).*  **Q62.** “I think you have probably always got that little bit more fear if you are taking somebody who isn’t on dialysis and *putting them on to* dialysis” *(Cardiology consultant, Trust 3).*  **Q63.** “we get one opportunity to talk to a cardiologist of a day, then we can talk to a cardio reg, depends on their seniority and their experience of dealing with renal cardiac patients as to what they’ll do. They might go off and talk to a consultant, that information might come back a number of hours later, things have changed by then, or decisions need to be made. It's difficult. I think that multidisciplinary team approach where a cardiologist and the renal team liaise together would make much more sense from the patient’s perspective.” *(Acute & emergency medicine consultant, Trust 1).*  **Q64**. “the patient 9 times out of 10 ends up with a coronary angiogram anyway and yet we do scans in between, things just take a lot longer than perhaps they should” *(Nephrology consultant, Trust 1).*  **Q65.** “we don't want to offer them surgery because of high risk, but they don't want to offer them stents because they're also high-risk for stents.” *(Cardiac surgical registrar, Trust 3).* |
| CKD; chronic kidney disease, HD; haemodialysis; PD; peritoneal dialysis.  * The term CKD is used here to refer to any patient with CKD who is not receiving a form of kidney replacement therapy. We appreciate that in practice, all individuals using kidney replacement therapies also have CKD. | | |
