# Supplementary material for: How are treatment decisions for myocardial infarction made in the presence of advanced kidney disease? A qualitative study in the UK
Source: BMJ Open. 2025 Oct 20;15(10):e106617. doi: 10.1136/bmjopen-2025-106617 (PMC12542582; doi:10.1136/bmjopen-2025-106617)
Supplement: Supplementary file 2 [file bmjopen-2025-106617.reviewer_comments.pdf]

PEER REVIEW HISTORY

BMJ Open publishes all reviews undertaken for accepted manuscripts. Reviewers are asked to complete a checklist review form and are provided with free text boxes to elaborate on their assessment. These free text comments are reproduced below.

ARTICLE DETAILS

Title (Provisional)

How are treatment decisions for myocardial infarction made in the presence of advanced kidney disease? A qualitative study in the UK.

Authors

Scott, Jemima; Selman, Lucy; Caskey, Fergus; Johnson, Thomas; Ben-Shlomo, Yoav; Graham-Brown, Matthew P; Bailey, Phillippa K

VERSION 1 - REVIEW

**Reviewer**	**1**
**Name**	**Townend, Jonathan**
**Affiliation**	**University of Birmingham, Cardiology, Cardio-Renal Group**
**Date**	**15-Jul-2025**
**COI**	**None**

---

1. The paper does not discriminate between NSTEACS in which treatment strategies can be considered and discussed over a period of hours or even days and STEMI which requires emergency revascularisation in almost all without time for discussion. This will have a big influence on the results. The authors need to define the type of 'MI' when talking to clinicians and differentiate between the two when analysing and discussing their data.

2. Similarly, most cardiologists would have widely differing attitudes and management plans for patients with stage 3 CKD (very common, most cardiologists would not adjust treatment much I suspect) and those with advanced CKD (stage 4 and 5). Attitudes and concerns for patients with 'kidney failure' on RRT or with a transplant will differ again. I dont think it is sensible or appropriate to lump together all of these patients as 'CKD' and to talk about results in common.

3. The number of patients was low and there were only 3 with CKD rather than kidney failure. This must limit the conclusions drawn.

4. I am not clear why the responses of clinicians were considered as a group. I wouid have expected that the responses of cardiologists and nephrologists might differ considerably.

5. While data are still lacking, there is consensus from trials and in guidelines (eg ESC ACS 2023) that patients with CKD of all severities benefit from early reascularisation compared to medical therapy. it is against this standard, that the responses should be discussed.

6. The authors should consider a revised paper examining cardiologists and nephrologists attitudes separately to patients with NSTEACS (excluding STEMI) and with advanced CKD (stage 4 and 5) or kidney failure.

**Reviewer**	**2**
**Name**	**Finderup, Jeanette**
**Affiliation**	**Aarhus Universitetshospital, Renal Medicine**
**Date**	**31-Jul-2025**
**COI**	**None**

How are treatment decisions for myocardial infarction made in the presence of advanced kidney disease? A qualitative study. - A clinically relevant study, which covers a significant knowledge gap.

Some areas for improvement:

The background lacks a rationale for conducting a study that interviews both patients and clinicians, particularly why patients are being interviewed.

The context of the study has not been described in the methods section. More information is needed about how this specific condition is treated and managed in the included trusts.

There are no references to any data collection methods. The interview guide for patients is missing.

The data analysis section does not describe how you handled the two groups of data. Did you analyse data from the two groups separately and then combine them, or what?

You report the number of participants invited and the number accepted, but not how many declined from the clinician group and how many from the patient group.

More information is need about the distribution of participants from each site, given that this is a multi-site study.

Some of your themes are only based on data from the clinicians. Then why use data from both groups of participants? In general, looking at quotations, your patient participants are underrepresented.

VERSION 1 - AUTHOR RESPONSE

**Comment**	**Response**

**Reviewer 1: Professor Jonathan Townend**	
The paper does not discriminate between NSTEACS in which treatment strategies can be considered and discussed over a period of hours or even days and STEMI which requires emergency revascularisation in almost all without time for discussion. This will have a big influence on the results. The authors need to define the type of 'MI' when talking to clinicians and differentiate between the two when analysing and discussing their data.	Thank you. We agree that there are marked differences in the treatment timescales and degree to which care pathways are protocolised between NSTEACS and STEMI. We have previously undertaken observational analyses of ACS care in England and found treatment disparities between people with and without CKD not only amongst those with NSTEACS but also following STEMI(1). Explaining these findings is challenging given that kidney function is frequently not known prior to a decision regarding invasive management being made.<br><br>We were able to specifically raise these findings in our interviews with clinicians, asking for their thoughts on how we could explain such disparities. Regarding this (sub)topic and several others (such as the ease of treatment decision-making and who was involved), we did differentiate between NSTEAC and STEMI when talking to clinicians.<br><br>With regards to our analysis, some of the themes identified were influenced by ACS type (such as “Differential risk to organs facilitates decision-making”). Clinicians perceived treatment decision-making to be easier in situations where the risk to one organ (or life), such as would frequently be the case in STEMI, significantly outweighed that to another organ, such as the kidney. A few of the quotations refer specifically to ACS type in this regard (for example Q49), but not all. This may reflect the fact clinicians were thinking beyond the binary classification of STEMI vs. NSTEACS to the underlying drivers of decision-making (for example, why treatment decision-making is simpler in STEMI).<br><br>The differentiation between NSTEACS and STEMI was mentioned less frequently in relation to some other themes, such as the importance of relationships between different specialties. It is possible that this does have a greater influence on treatment decision-making where there is time for collaboration, i.e. decisions regarding invasive care in NSTEACS. This wasn’t raised in the interviews however, so we can only postulate.<br><br>Overall, we agree that there exist differences in treatment decision-making between NSTEACS and STEMI care. It is interesting that this didn’t come out more strongly in our interviews, and we have added a note regarding this to the limitations section of our discussion (pg. 15); “Some of the themes, such as

	“Effective relationships are dependent on trust” may be less relevant to time-critical care such as that for ST-elevation MI”.
Similarly, most cardiologists would have widely differing attitudes and management plans for patients with stage 3 CKD (very common, most cardiologists would not adjust treatment much I suspect) and those with advanced CKD (stage 4 and 5). Attitudes and concerns for patients with 'kidney failure' on RRT or with a transplant will differ again. I don’t think it is sensible or appropriate to lump together all of these patients as 'CKD' and to talk about results in common.	Again, we agree with this view, and this qualitative study was able to explore these differences.<br><br>The patients included in our interview study had CKD stage 4 or 5 (some of whom had a functioning transplant or received dialysis). Our interviews with clinicians were focussed on this group of individuals, although comparisons to those with milder CKD were often brought up spontaneously.<br><br>People with CKD stages 4 and 5 are frequently grouped together for pragmatic reasons in observational work. We agree that this is frustrating, as it often misses the nuance of differences in decision-making in sub-groups. In a systematic review of global use of invasive ACS management in people with and without CKD that we recently undertook, we found, for example, a signal that individuals receiving dialysis may be more likely to receive coronary angiography than those who have CKD4/5 but have not started dialysis. This would be consistent with our clinical experience that clinicians are most anxious about invasive management in those that have some residual kidney function remaining “at risk” of contrast-associated AKI etc.<br><br>The benefit of this qualitative work was that we were able to explore the nuances of ACS treatment decision-making. One of the questions we asked in the interviews was “would it make a difference if the patient were to have CKD 4/5/be in receipt of dialysis”, to enable us to explore how severity of CKD and/or receipt of specific forms of KRT influenced decisions.<br><br>Similar to the valid point you raised regarding the differences between NSTEACS and STEMI decision-making, CKD severity/KRT form appeared to be relevant to some themes, but not others. For example, collaborative treatment decision-making appeared important irrespective of the degree of kidney impairment. In contrast, in theme 6 (“Individual perception of risk and benefit”), KRT choice appears to have a central role in decision-making.
The number of patients was low and there were only 3 with CKD rather than kidney failure. This must limit the conclusions drawn.	We agree that ideally we would have had better representation of patients with CKD who were not receiving KRT, and have added this to our limitations section. However, responses of our patient participants

	did not appear to vary particularly between those receiving KRT and those who were not. In fact, whilst the patient data was extremely interesting in itself, the main theme highlighted from these interviews was the minimal contribution that patients made to inpatient decision-making overall. We therefore believed that increasing our numbers of patient participants further would not influence the results of our study.
I am not clear why the responses of clinicians were considered as a group. I would have expected that the responses of cardiologists and nephrologists might differ considerably.	Thank you for raising this. You are quite right that we could have chosen to analyse the results of each clinician group separately. Our interest, however, was the similarities and differences in decision-making across specialties. Overall, we were surprised by the degree of similarity in decision-making between specialties. Differences between individual clinicians appeared more marked than between specialties as a whole.<br><br>Two areas that did appear to be influenced by specialty were, i) estimation of prognosis of individuals with CKD (non-nephrologists appeared more pessimistic) and ii) interpretation of the evidence base.<br>We found it fascinating that, despite access to the same body of published research, the specialty of the clinician appeared to influence how this evidence was interpreted – and hypothesise that it is the differences in clinical experience, determined by the specialty in which individuals work, which influences this.<br><br>We also noted that nephrologists appeared to believe cardiologists over-estimated the risks of coronary angiography and PCI in individuals with CKD, whilst cardiologists believed the same of their cardiac surgical colleagues. Thus it is more likely that specialties' estimation of risk is determined by whether they undertake the relevant procedure, rather than specialty per se.
While data are still lacking, there is consensus from trials and in guidelines (eg ESC ACS 2023) that patients with CKD of all severities benefit from early revascularisation compared to medical therapy. It is against this standard, that the responses should be discussed.	We agree, and it will be interesting to see whether this change in stance of the ESC guidelines will result in changes in clinical practice. From our data regarding how guidelines and protocols are used, we remain suspicious that a change in guideline will not (rapidly) alter practice, however. Clinicians, in particular those who had been practising for longer, spoke frequently about relying on their own clinical experience and that of colleagues, above written documents. It was suggested that this was more often the case when there was any lack of clarity/grey zone within a guideline.

	To highlight this point, and ensure that appropriate reference to the ESC 2023 guidelines has been made, we have added a sentence into our discussion (pgs. 13-14) which now reads: "...It remains to be seen therefore, whether the updated recommendation in the 2023 European Society of Cardiology guidelines to "apply the same diagnostic and therapeutic strategies [for ACS] in patients with CKD as in patients without kidney disease" will significantly alter clinical practice [14]."
The authors should consider a revised paper examining cardiologists and nephrologists attitudes separately to patients with NSTEACS (excluding STEMI) and with advanced CKD (stage 4 and 5) or kidney failure.	While we appreciate this suggestion for future research, this was not the aim of the current paper. As discussed above, in this study we were interested in the similarities and differences across specialties, and we have highted in the results where either specialty or CKD severity influenced the themes identified.
**Reviewer 2: Dr Jeanette Finderup**	
The background lacks a rationale for conducting a study that interviews both patients and clinicians, particularly why patients are being interviewed.	Thank you for highlighting this. It is possible that disparities in MI care between people with and without CKD are driven by difference in patient treatment choices. This is important, as it would represent an equitable reason for an inequality.<br><br>In our background we initially wrote, "It is unclear .... to what extent decision-making is shared (between patients and clinicians and between specialists)..." pg. 4. For clarity, we have changed the sentence to: "It is unclear....to what extent decision-making is shared, whether decisions are being driven by patient wishes....", and hope this reads more easily.<br><br>The following paragraph states "This qualitative study was designed to understand how MI treatment decisions are made by people with CKD and the clinicians who care for them". This summarises the purpose of including patients with CKD as without their voice, we would be unable to determine the extent to which they are involved in treatment decisions.
The context of the study has not been described in the methods section. More information is needed about how this specific condition is treated and managed in the included trusts.	Apologies that this was unclear. We have now amended our wording of "hospital trusts" to "hospitals" (pg. 5). We have clarified that all hospitals from which participants were recruited hosted inpatient cardiology services and had percutaneous coronary intervention (PCI) capability, at least in working hours. We have specified the number of hospitals that, in addition, also had nephrology or cardiac surgery departments (3 and 2 respectively). We have not detailed the combination of the specialties available per trust as this could risk identification of the involved hospitals.

There are no references to any data collection methods. The interview guide for patients is missing.	We have included a section titled "Data Collection" (pg. 5). We specified that we conducted single in-depth semi-structured interviews with each participant, either in-person or by videoconference or telephone according to participant preference. We added that the interviews were conducted by JS, followed flexible topic guides (separate for patient and clinician participants) and were audio-recorded. We also mentioned how the topic guides were developed and that we collected demographic data at the time of interview. The topic guides are now included as supplementary material.
The data analysis section does not describe how you handled the two groups of data. Did you analyse data from the two groups separately and then combine them, or what?	Thank you for highlighting this.<br><br>We have added the following sentence to our section on data analysis (pg. 6); "Data from patient and clinician participants were analysed separately and the findings combined by identification of over-arching themes which captured both coding frameworks".
You report the number of participants invited and the number accepted, but not how many declined from the clinician group and how many from the patient group.	We have added this detail in (please see pg. 7 ("37 patients and 73 clinicians"), and also amended our total participant figure which was erroneously low at 33 (and should have been 47).
More information is need about the distribution of participants from each site, given that this is a multi-site study.	Thank you for this suggestion. We have added an extra tale to our supplement to provide this information (e. Table 7). We have not differentiated into either participant type or clinician specialty as, due to the specifics of the sites recruited, this could result in Hospital Trusts being identified. We have explained this further in our letter to the Editor.
Some of your themes are only based on data from the clinicians. Then why use data from both groups of participants? In general, looking at quotations, your patient participants are underrepresented.	The purpose of this study was to determine how MI treatment decisions are made in England, for/by people with CKD and thus to lead us one step further to understanding why disparities in MI care exist between people with and without CKD and whether or not such disparities are equitable.<br><br>We based our participant selection on the assumption that MI treatment decisions would be, to at least some extent, shared between patient and clinician. We used an inclusive recruitment strategy to reflect this. The alternative would have been to assume that patients do not contribute to treatment decisions and to exclude them, or to base our reflections on the importance of the patient voice only on clinicians' perceptions, which we felt would be biased.

	Through analysing the patient data, we found that people with CKD, at present, perceive themselves to contribute extremely little to inpatient MI treatment decision-making (pls see pg. 7, Results: “Theme 1: Limited patient involvement in treatment decisions”). This is an important finding in itself which relied on the inclusion of patient voices to help us explain the process of treatment decision-making. It was corroborated by the majority of clinicians, although some described involving patients in shared decisions (especially when they themselves were unclear regarding the “best” option), and the challenges they faced in doing so (lack of time/lack of evidence etc).<br><br>This lack of patient involvement is decision-making is reflected in the Results, with patient data relevant to some themes (“Effective relationships are dependent on trust”/”Influence of kidney replacement therapies on perceived risks”), but not present in others.

References

1. Scott JK, Johnson T, Caskey FJ, Bailey P, Selman LE, Mulla A, et al. Association between kidney function, frailty and receipt of invasive management after acute coronary syndrome. Open Heart. 2024;11(2):e002875.

VERSION 2 - REVIEW

**Reviewer**	**1**
**Name**	**Townend, Jonathan**
**Affiliation**	**University of Birmingham, Cardiology, Cardio-Renal Group**
**Date**	**19-Aug-2025**
**COI**	

Thank you for the responses and amendments. The responses were illuminating but I am a little disappointed to find that they are not much represented in the amendments to the MS.

Specifically:

1. For the patient group, the STEMI/NSTEMI numbers should be given so we are clear about what sort of 'MI' and therefore whether emergency or urgent treatment was offered. It is important to know (categorically) whether STEMI and NSTEMI patients were treated

differently because of the presence of CKD. (The general reader may need reminding about the 2 categories of type 1 MI and the differences in standard treatment approach)

2. The NSTEMI/STEMI issue and its influence on treatment decisions should be discussed much more clearly. In section 6 the authors refer to a 'life threatening MI'! This is near nonsensical; all MIs are life threatening as the mortality from STEMI and NSTEMI does not differ greatly. Do you really mean STEMI?

3. If the difference in treatment decisions between patients on and off KRT was specifically addressed, where are the data in the results?

4. The very small number of patients with CKD but not kidney failure requiring KRT (3) is said to have been added to the limitations section but this does not appear to have been inserted, In essence, the patient results are very largely derived from those on KRT and no strong inferences should be drawn from those with early stage CKD.

5. I would like to see a comment in the discussion on differences and similarities in approach to patients with CKD between nephrologists and cardiologists as provided in the response.

6. The existing ESC guidance on ACS/CKD should be alluded to at the beginning of section 3 of the results. The scepticism and lack of reliance on (or knowledge of?) these guidelines is highly surprising to me as ESC guidelines are intensively used and taught to trainees (at least in cardiology). There is an evidence base for these guidelines which the clinicians appear to either be unaware of, ignore or dismiss. A stronger comment in the discussion would be appropriate.

**Reviewer**	**2**
**Name**	**Finderup, Jeanette**
**Affiliation**	**Aarhus Universitetshospital, Renal Medicine**
**Date**	**20-Aug-2025**
**COI**	

Thank you for improving your paper.

In the section about data collection, I have not been able to identify any references for a data collection method.

VERSION 2 - AUTHOR RESPONSE

**Comment**	**Response**
**Reviewer 1: Professor Jonathan Townend**	
Thank you for the responses and amendments. The responses were	We are sorry to hear that you are not satisfied by our responses. Our research group undertakes observational in addition to

illuminating but I am a little disappointed to find that they are not much represented in the amendments to the MS. Specifically:<br><br>For the patient group, the STEMI/NSTEMI numbers should be given so we are clear about what sort of 'MI' and therefore whether emergency or urgent treatment was offered. It is important to know (categorically) whether STEMI and NSTEMI patients were treated differently because of the presence of CKD. (The general reader may need reminding about the 2 categories of type 1 MI and the differences in standard treatment approach)	qualitative work, and whilst many of your comments are highly relevant to the former type, here we report qualitative work rather than, for example, a quantitative observational matched cohort study. Thus our priorities were understanding how clinicians and patients make treatment decisions (rather than what treatment decisions they make) and representing the (overt and subliminal) voices of our participants. We wished to compare and contrast between patient groups, but without imposing our own categorisations – such as between NSTEMI and STEMI. Qualitative research can rarely tell the reader any fact categorically – this is the role of observational and trial evidence.<br><br>In response to your request for STEMI/NSTEMI numbers within the patient population, we are afraid this is not possibly to provide as these data were not collected. Demographic and clinical data were collected directly from patient participants – a trivial minority make any remark on their MI type in their responses, strongly suggesting that MI type did not impact patient decision-making regarding treatment. Even with the MI types documented, we would be unable to make any categorical statement regarding whether STEMI and/or NSTEMI patients were treated differently because of their CKD. Not only because we have no "control" group (control groups being used rarely if ever in qualitative research), but also because our numbers of patients are too few to draw definite numerical conclusions. Fortunately, this was not the aim of the research. Our previously published international systematic review and meta-analysis and English observational cohort shed light on numerical differences on MI management between those with and without CKD.<br><br>We appreciate that current guidelines recommend very different treatment pathways for those with STEMI versus those with NSTEMI and have very happily added a few sentences along those lines at the end of the 1st paragraph of our introduction ("Such variation in care impacts not only individuals experiencing non-ST elevation MI (NSTEMI) but also those with ST-elevation MI (STEMI) for whom highly protocolised emergency intervention is advocated within international guidelines[4-7].)
The NSTEMI/STEMI issue and its influence on treatment decisions should be discussed much more clearly. In section 6 the authors refer to a 'life threatening MI'! This is near nonsensical; all MIs are life threatening as the mortality from STEMI and NSTEMI does not differ	Whilst clinical guidelines categorise MIs in multiple different ways, the use of specific terminology (such as NSTEMI/STEMI) by clinicians in these interviews was reasonably rare, which is of interest in itself. One explanation would be that MI "type" doesn't influence decision-making as much as the perception of the MI being 'life-threatening/organ-threatening'.<br><br>With regards to your comment about the 'nonsensical' nature of the phrase "life threatening MI", this is an exact quotation

greatly. Do you really mean STEMI?	from one of our participants (in this case a consultant cardiologist). In qualitative research the language of the participants is used to ensure that the nuance of their ideas and beliefs is not lost. This highlights the value of qualitative work: reporting the terminology, language, and understanding of a diverse group of practising clinicians that may differ from 'academic experts' who 'write the guidelines'.
If the difference in treatment decisions between patients on and off KRT was specifically addressed, where are the data in the results?	These data are described in theme 6. The relevant subtheme is presented on page 11 and is titled "Influence of kidney replacement therapies on perceived risks". The relevant text is copied below:<br><br>**Influence of kidney replacement therapies on perceived risks**<br>The (planned) receipt of KRTs influenced decision-making by all participants. For many clinicians, the availability of KRTs encouraged them to prioritise the heart above the kidney (Q51). Individuals with or awaiting a kidney transplant were thought appropriate for invasive management as they had been deemed fit for surgery and required optimal cardiac function to survive this (Q52). Patients suspended from the transplant list indicated they would undergo any intervention to be relisted (Q53). In contrast, perceptions of the "meaning" of dialysis differed. Some believed dialysis represented "active" therapy, suggesting these patients should receive invasive MI management (Q54). Conversely, a few non-nephrologists equated receipt of dialysis with significant frailty and thus suitability for medical MI management only (Q55). One patient believed clinicians considered all CKD patients "on [their] way out" (Q56).
The very small number of patients with CKD but not kidney failure requiring KRT (3) is said to have been added to the limitations section but this does not appear to have been inserted, In essence, the patient results are very largely derived from those on KRT and no strong inferences should be drawn from those with early stage CKD.	We apologise for this omission, and have ensured that the appropriate text has now been added to our limitation section (pg 15. "Patient participants were predominantly those receiving KRTs.)<br><br>As per the title, this qualitative research study addressed treatment decision-making in those with advanced CKD. We do not attempt to infer anything regarding the management of those with early-stage kidney disease.<br><br>In this study we aimed to achieve a diverse sample, rather than a population representative sample which is associated with quantitative research. Qualitative studies seek to provide in-depth and diverse insights into a phenomenon and do not attempt to generalise the results to a population of interest(1).<br><br>Most qualitative studies are necessarily small scale to provide depth and contextualised detailed information, and thus the

	criterion of generalisability (external validity) cannot be applied. Instead, transferability is the extent to which the concepts and theories are relevant to other settings. Researchers can compare their results with studies conducted in different healthcare contexts, regions, or populations; position their findings with other theoretical frameworks; and describe the study setting and participant characteristics in detail so readers can judge the transferability of the findings to their own context(2).
I would like to see a comment in the discussion on differences and similarities in approach to patients with CKD between nephrologists and cardiologists as provided in the response.	Thank you for suggesting this addition. We have stretched our word count to enable this, but having done so we think it does contribute very nicely to the discussion. The changes are found on pages 13-15 and summarised below:<br><br>The first paragraph of the discussion now includes the sentence – "The potential to "cause" kidney failure was the most feared outcome amongst non-nephrologists," however in general, differences in treatment decision-making appeared more marked between individual clinicians than between specialties."<br><br>On pg 15 we have added the following paragraph:<br>"Variation in treatment decision-making appeared greater between individual clinicians than between specialties. Specialty did appear however, to influence decisions regarding i) estimation of prognosis of individuals with CKD (non-nephrologists appeared more pessimistic) and ii) interpretation of the evidence base. Despite access to the same body of published research, clinicians' views on clinical concerns such as the risk and impact of contrast-associated nephropathy varied widely between specialty. As suggested previously, it is possible that clinical experiences (personal and those of trusted colleagues) influence clinicians' choice and interpretation of research papers."<br><br>Our only concern in adding this text is the extension in length of the discussion.
The existing ESC guidance on ACS/CKD should be alluded to at the beginning of section 3 of the results. The scepticism and lack of reliance on (or knowledge of?) these guidelines is highly surprising to me as ESC guidelines are intensively used and taught to trainees (at least in cardiology). There is an evidence base for these guidelines which the clinicians appear to either be unaware of, ignore or dismiss. A	I would add reference to the ESC guidance he mentions, but this shouldn't be added to the results if it wasn't mentioned by the participants, but do add it to the discussion.<br><br>Similarly, we were surprised by clinicians' reflections on their use of national/international guidelines. However, we feel these findings are useful to both guideline-writers and those trying to understand why disparities between clinical practice and such guidelines exist.<br><br>We have not altered our results as here we discuss clinicians' perceived use of a wide range of guidelines and protocols. We

stronger comment in the discussion would be appropriate.	did not find that clinicians responses varied between specific guidelines.<br><br>In the discussion, the discrepancy between evidence/guidelines and clinical practice is now mentioned on two occasions following the addition of the paragraph on differences in decision-making between specialties (as above), and the previous wording as follows: "In general, despite the concept of evidence-based medicine being widely accepted, there remained scepticism of guidelines or epidemiological data especially in the light of personal adverse experiences or anecdotes. This highlights the challenges in translating evidence into real world clinical management. It remains to be seen therefore, whether the updated recommendation in the 2023 European Society of Cardiology guidelines to "apply the same diagnostic and therapeutic strategies [for ACS] in patients with CKD as in patients without kidney disease" will significantly alter clinical practice [14].
**Reviewer 2: Dr Jeanette Finderup**	
Thank you for improving your paper. In the section about data collection, I have not been able to identify any references for a data collection method.	Our apologies for mis-interpreting your previous comment. We have now added the following references:<br><br>Leavy, Patricia (ed.), The Oxford Handbook of Qualitative Research, 2nd edn, Oxford Handbooks (2020; online edn, Oxford Academic, 2 Sept. 2020), https://doi.org/10.1093/oxfordhb/9780190847388.001.0001, accessed 11 Sept. 2025.<br><br>Irani E. The Use of Videoconferencing for Qualitative Interviewing: Opportunities, Challenges, and Considerations. Clin Nurs Res. 2019 Jan;28(1):3-8. doi: 10.1177/1054773818803170. PMID: 30470151.

VERSION 3 - REVIEW

**Reviewer**	**1**
**Name**	**Townend, Jonathan**
**Affiliation**	**University of Birmingham, Cardiology, Cardio-Renal Group**
**Date**	**15-Sep-2025**
**COI**	

Improved in response to review, remaining problems have been discussed appropriately
